# Research collaboration in groups and networks: differences across academic fields

**DOI:** 10.1007/s11192-017-2497-5

**Published:** 2017-09-04

**Authors:** Svein Kyvik, Ingvild Reymert

**Affiliations:** NIFU (Nordic Institute for Studies in Innovation, Research and Education), Oslo, Norway

**Keywords:** Research groups, Research collaboration, Academic fields, Norwegian research universities, Publication output

## Abstract

The purpose of this paper is to give a macro-picture of collaboration in research groups and networks across all academic fields in Norwegian research universities, and to examine the relative importance of membership in groups and networks for individual publication output. To our knowledge, this is a new approach, which may provide valuable information on collaborative patterns in a particular national system, but of clear relevance to other national university systems. At the system level, conducting research in groups and networks are equally important, but there are large differences between academic fields. The research group is clearly most important in the field of medicine and health, while undertaking research in an international network is most important in the natural sciences. Membership in a research group and active participation in international networks are likely to enhance publication productivity and the quality of research.

## Introduction

Broadly speaking, most research is undertaken as a collaborative effort in *groups* and *networks* of scientists. The rationale is that two or more people can do better work than if they work independently of each other. Collaboration in research can take different forms, from giving advice to colleagues to working closely together. Collaboration can be undertaken between colleagues in a university department, between a staff member and peers in other departments, in other universities and research institutes, in industry, and in research establishments in other countries. Collaboration can take place between two individual researchers or between many scientists as members of large teams. Collaboration can be a hierarchical relationship, like the one between a professor and a doctoral student, or a mutual relationship between two or more colleagues of equal status. And collaboration can take place voluntarily between colleagues who share the same interests and go well together, or be more or less forced upon researchers, like in large-scale programmes initiated by research councils where funding is dependent on the willingness to join forces with other scientists, often in other disciplines. Collaboration with other scientists can be stimulating and enriching, but also problematic and conflict-ridden. Tensions and conflicts can relate to choice of research methodology, interpretation of data, and writing style, not to mention how each individual contribution should be credited.

Collaboration in *groups* has long traditions in the experimental sciences, and has been called the engine of productivity in research and of effective graduate training (Etzkowitz 1992), while collaboration in research *networks* is a parallel and complementary, and to some extent a competing organisation principle to the research group in the university department or in an industrial laboratory. Commonly, research networks differ from research groups due to their greater flexibility and less bureaucracy (Leite and Pinho 2017). Collaboration in groups and networks has become increasingly common, as witnessed by the growth in co-authorship of scientific papers (Leydesdorff and Wagner 2008). The reasons are multiple; the need for a critical mass of people with complementary skills and expertise (Ziman 1987; Heinze et al. 2009), the growing ease of travel and the introduction of the internet (Katz and Martin 1997), the need for more interdisciplinary research (Lee and Bozeman 2005), political initiatives for more research co-operation and cost-sharing (Katz and Martin 1997), the self-interest of researchers to link together in search of rewards, reputation, and resources (Wagner and Leydesdorff 2005) or to further an academic career (Rijnsoever et al. 2008), and various non-scientific individual motivations like the opportunity to travel and maintain friendships (Melin 2000).

Academic studies of research groups abound, but are most often confined to the STEM fields. The first extensive investigation of research groups was undertaken in the 1970s (Andrews 1979). Communication and collaboration between team members, the role of the leader, research experience, and group size were identified as important factors to explain variation in group productivity. These factors have been examined in a large number of follow-up studies: Size and productivity (Cohen 1991; Seglen and Aksnes 2000; Guimerà et al. 2005; Heinze et al. 2009; Wheelan 2009; Kenna and Berche 2011; Maaike et al. 2015), cooperation within groups (Andrade et al. 2009), the role of the leader (Nagpaul and Gupta 1989; Hemlin 2006; Pudovkin et al. 2012; Maaike et al. 2015), and the groups’ importance for PhD-students (Meschitti and Carassa 2014). A review of the literature on research groups indicates that belonging to an established research group leads to higher scientific production (e.g. Martin-Sempere et al. 2002), while not belonging to a consolidated research team represents a handicap in terms of publishing in top international journals (Rey-Rocha et al. 2002). Furthermore, there is evidence that group leaders have more publications and are more often cited than the average group member (Lazega et al. 2008).

There are also an increasing number of studies on scientific collaboration networks (see e.g. Lazega et al. 2008), many of which take social network theory as a starting point. The different ‘actors’ are connected to each other through ‘ties’ of different kinds which can be strong or weak, hierarchical or equal, geographically close or distant, etc. (Scott 2000). According to Castells (2000) social networks are gradually replacing hierarchical forms of organization in their specific realms of activity. Research collaboration in networks is no exception to this general trend and has become much more important over time with the strongly improved conditions for physical and virtual communication (Glänzel and Schubert 2004). Likewise, Adams (2012, 335) has stated that “a fundamental shift is taking place in the geography of science. Networks of research collaboration are expanding in every region of the globe”. A review of the relevant literature indicates that research collaboration in international networks enhances the productivity of individual scientists (Kyvik and Larsen 1994; Van Raan 1998; Martin-Sempere et al. 2002; Barjak and Robinson 2007; Abramo et al. 2011) and the quality of research (Adams 2012).

The research done in groups and networks is often closely related. Groups to an increasing degree collaborate across institutional and national borders. According to Adams (2013, 557) research has progressed through three ages: the individual, the institutional and the national, and is now entering a fourth age driven by international collaborations between elite research groups. Furthermore, group members can be connected to various networks. A partial reason is that groups are not stable over time; members (particularly graduate students and postdocs) move on to other groups to further their careers and obtain tenure, while keeping in touch and continue collaborating with their former supervisors and colleagues (Jacob and Meek 2013). Hence, groups constituted by individuals with disparate sets of collaborators are more likely to draw from a more diverse reservoir of knowledge (Guimerà et al. 2005).

While many studies have examined collaboration in groups and networks separately, we are not aware of any study that has aimed to investigate the relative importance of groups and networks for individual researchers. What does membership in a research group actually imply for the research undertaken by individual members? To what extent do members undertake most of their research within the frame of the group in the department versus in networks? To what extent does collaboration in groups and networks enhance productivity and quality of research? Are there differences between academic fields in these respects? In order to explore these issues, the four major Norwegian research universities are used as a case.

Although the notions of research groups and research networks should be intuitively clear, in practice it is often difficult to distinguish and operationalize groups and networks for measurement purposes. The standard methodology is to use bibliometric databases to identify co-authorship patterns within and across institutions based on author addresses, but it is a challenge to distinguish between members of the core group and external participants in a network relationship. Hence, several studies on scientific networks do not distinguish between collaboration in groups and networks (e.g. Newman 2001). In this respect, this paper differs from prior studies because it is based on information from academic staff themselves on the extent to which they conduct their research within a formal group or within a network. In this study, groups are institutionally based while networks cross institutional boundaries.

*Formal research groups* have now been introduced in all fields as subunits within departments (Vabø et al. 2016). In medicine and health, technology and the natural sciences, this policy for the most part has been a formalization of already existing groups, while in the humanities and the social sciences this development represents something new. An important reason is the creation of larger departments through mergers and a general growth in the number of academic staff, and the subsequent need for organizing research and teaching within the frames of smaller subunits. The purposes of the formalization of these groups are to enhance research collaboration, strengthen research leadership, create good scientific and social environments of academic staff and doctoral students, contribute to the implementation of research strategies, and establish an organizational platform to increase external research funding. This has also been part of a trend where research and education increasingly have been organized across academic fields (Michelsen and Vabø 2014; Vabø et al. 2016).

While it should be clear to all respondents whether they are members of a formal group, information on collaboration in formal and informal research networks is based on the actors’ individual interpretations of what constitutes a network and the strength of their participation, and not on bibliometric data on co-authorship.

The purpose of this paper is to provide a macro-picture of collaboration in research groups and networks across all academic fields in these universities. Another purpose is to examine the relative effect of conducting research within groups versus in networks for scientific and scholarly publishing. To our knowledge, this is a new approach, which may provide valuable information on collaborative patterns in a particular national system, but of clear relevance to other national university systems.

## Data and methods

Data are drawn from a survey in 2013 to professors and associate professors in permanent positions at the four major traditional research universities in Norway; the University of Bergen, the University of Oslo, the University of Tromsø, and the Norwegian University of Science and Technology. These four institutions are much more research oriented than the other newer universities (Reymert et al. 2015). The number of responses included in this sample is 1481, and the response rate is 50.2%. The sample of researchers who responded to the survey is representative in respect of gender, age, and academic field (Waagene and Reymert 2015). This unique survey enables the investigation of the extent to which membership of a research group implies that the members conduct their research within the group, the extent to which they collaborate within networks of research colleagues, or work independently.

In the survey, we asked the respondents to which degree they use different ways of conducting research: (a) alone, (b) with colleagues in the department not affiliated to a formal research group, (c) in a formal research group in the university, (d) in a national research centre, (e) in an interdisciplinary centre, (f) in a national network, or (g) in an international network. For each of these categories the respondents were given three alternatives: “to a large degree”, “to some degree” or “to a minor degree/not at all”.

These different alternatives are, however, not mutually exclusive. Seven out of ten of the respondents reported one single way of doing research “to a large degree”, while more than 20% reported two alternatives “to a large degree”, and the rest different combinations of three or more options. The various responses produced a vast number of combinations, and we have undertaken several adjustments to be able to group the researchers into manageable categories.

Firstly, those who answered “in a national research centre”, or “in an interdisciplinary research centre” were classified as conducting research “in a formal research group”. Secondly, we excluded the category ‘collaborating in a national network’, since only 17 persons responded that they undertake research “to a large degree” only in this way. Those who work alone “to a large degree” and collaborate in various ways “to a large degree”, were included in the respective categories of researchers collaborating with other people, leaving the group doing research “alone” as a pure group of solitary researchers. Researchers who reported to collaborate “to a large degree” informally with colleagues in the institution and also in a formal research group were included in the category “formal research group”. Finally, those who responded that they in addition to doing research “to a large degree” in international networks also undertake research “to large degree” in other ways were classified as international collaborators. These adaptions were done because we are particularly interested in the effects of groups and networks on performance in research.

The analysis of the publication output of academic staff is based on a bibliographic database that has been developed in Norway as a common and complete documentation system for all scientific and scholarly publications (Cristin). This database has complete coverage of all peer-reviewed scientific and scholarly publications, including journal articles, monographs, book chapters and conference series in all fields of research (Sivertsen 2016). Different publications give different publication points at two different levels (see Table 1). Level 1 is the ordinary scientific publication channel, while Level 2 is confined to prestigious journals and publishers (Table 2).

**Table 1:** Publication points for different publications at different academic levels

	Level 1	Level 2
Articles in scientific journals	1	3
Chapters in anthologies	0.7	1
Monographs	5	8

**Table 2:** Distribution of publication points and publications at Level 2 among professors and associate professors at the four major research universities in Norway, 2011–2013

Dependent variables	Publication points	Publications at Level 2
Minimum value	0	0
Maximum value	23.4	1
Mean	2.987	27,1%
SD	3.108	0.274
Mean among those who have published	3.336	47,2%
SD	3.103	0.189
Share of value zero/non-publishers (frequency/%)	155 (10%)	630 (43%)

At the time this study was conducted, if there were more than one author of a publication, the publication points were shared between the authors. Hence, the amount of publication points was affected by the number of co-authors. Because it is much more common to be many co-authors of a paper in medicine than in the humanities; subsequently humanists tend to have more publication points than academics in medicine, even though medical scientists have more papers on their publication lists. One should therefore be careful to compare publication points across academic fields.

In the bivariate analyses, we use the total number of publication points in the 3-year period 2011–2013 as an indicator of research productivity, and the percentage of staff having publications at Level 2 in this period as an indicator of research quality.

The distributions of publication points and percentage of publications at Level 2 are very skew. 10% of the permanent academic staff do not have publication points, and 43% do not have publications at Level 2. On average, the academic staff have 3.0 publication points and 27% of their publications at Level 2 (see Table 3).

**Table 3:** The independent variables used in the regression analyses

Variable	Values	Frequency	Percent
Gender	Female = 0	445	30
	Male = 1	1036	70
Age	Less than 40 years	130	9
	40–49 years	368	24
	50–59 years	500	34
	60 years or more	483	33
Group membership	Non-member (reference category)	339	25
	Ordinary member	722	52
	Group leader	324	23
Rank	Professor	891	60
	Associate professor	590	40
Academic field	Humanities	339	23
	Social sciences	392	26
	Natural sciences	247	17
	Technology	219	15
	Medicine and health (reference category)	284	19
Ways of conducting research “to a large degree”	None of the ways	223	18
	Alone	286	24
	Informal collaboration	88	7
	Formal research group	268	22
	International network	154	13
	Informal/formal group and international network	189	16

To investigate the relative importance of being member of a research group on the way the researchers report to conduct their research, we have undertaken a logistic regression with dichotomous variables of ways of conducting research ‘to a large degree’ as dependent variables; alone, in a formal research group, in an international network, and in an international network in combination with informal or formal collaboration in the department.^1^ We use the humanities as reference category since this field differs most from the average, and because there are many researchers in this group which gives the greatest number of researchers in the references category. Age, gender, group membership, rank, and academic field are independent variables.

The *age* of academic staff is included because two prior surveys among Norwegian university staff found that older academics were less involved in international collaborative research than their younger peers (Kyvik and Olsen 2008). Likewise, *gender* is included as an independent variable because these surveys revealed that women to a lower extent than their male counterparts were part of international research collaborations (Kyvik and Teigen 1996. *Academic rank* is another variable that has shown to have explanatory power in analyses of international research collaboration; the higher the rank, the more academics work together across national borders (Kyvik and Larsen 1994). *Group membership* is included because several studies have shown that group leaders commonly have more publications and citations than the average researcher. Finally, *academic field* has proved to be an important variable in explaining individual differences in international collaborative research (Kyvik and Larsen 1994).

We have also undertaken regression analyses with the number of publication points and the percentage of publications at Level 2 as dependent variables. Since both these dependent variables are skewed, an OLS-regression without adjustments would be inappropriate. Hence, we have made three separate models; two logistic regressions for those who have and have not publication points, and have and have not publications at Level 2; and one model where we exclude the researchers who do not have publication points. We use the logarithm of the number of publication points as a dependent variable. This adjustment makes the dependent variables much more like a normal distribution, and an OLS-regression is an appropriate model to use. The interpretation of the coefficients would then be as changes in percentages and not in log-units as for the two other logistic models we use.^2^

As independent variables, we have included gender, age, academic rank, group membership (non-member, ordinary member, group leader), academic field, and the various ways of undertaking research ‘to a large degree’. The independent variables are operationalized in Table 3.

## Results

At the four major Norwegian research universities, eight out of ten of the permanent academic staff are members of a formal research group. The rate of membership varies between academic fields, with the highest percentages in medicine and natural sciences, and the lowest in the humanities where only six out of ten are members.

However, among the group members, only 26% conduct their research “to a large degree” in a formal research group, while 12% conduct their research “to a large degree” in an international network and 18% in an international research network in combination with informal or formal collaboration in the university (Table 3). Two out of ten report that they conduct their research to a large degree alone. Hence, being a member of a formal research group does not necessarily imply that most of their research is conducted within the group.

Among those who are group members, the relative importance of the research group differs much across fields (Table 4). Particularly in the humanities a large proportion of the staff conduct most of their research alone. In the humanities, only 10% of the group members conduct their research “to a large degree” in the group while 45% undertake their research “to a large degree” alone. In contrast, 42% of the members in medicine and health conduct their research “to a large degree” in a formal group, and only 7% conduct their research alone. We find the same difference between the two academic fields in how they are involved in international networks. While only 8% of the humanists conduct their research in an international network in combination with a group, 19% of the researchers in medicine and health do that.

**Table 4:** Percentages of group members who conduct their research in various ways to “a large degree”

	Members	Non-members	Humanities	Social science	Natural science	Technology	Medicine/health
Alone	20%	38%	45%	29%	9%	19%	7%
Informal collaboration	6%	11%	6%	6%	6%	9%	11%
Formal research group	26%	10%	9%	18%	25%	23%	42%
International network	12%	14%	17%	10%	21%	11%	5%
Informal/formal research group and an international network	18%	9%	8%	18%	19%	16%	19%
None of the ways	18%	18%	16%	20%	20%	23%	16%
*N* (total)	890	273	277	319	220	117	215

The research group is clearly most important in the field of medicine and health, while undertaking research in an international research network, as the only way of conducting research or in combination with formal or informal collaboration in the institution, is most important in the natural sciences. The results indicate that the traditional field differences persist, even though formal research groups are introduced in all academic fields.

An important question is whether those who are not members of a formal research group differ from the group-members in ways of undertaking research. Are research networks an important alternative to group membership for these academic staff members? Among the non-members, the majority undertake their research alone (38%), while a lower share engage in an international research network (22%).

**Fig. 1:**
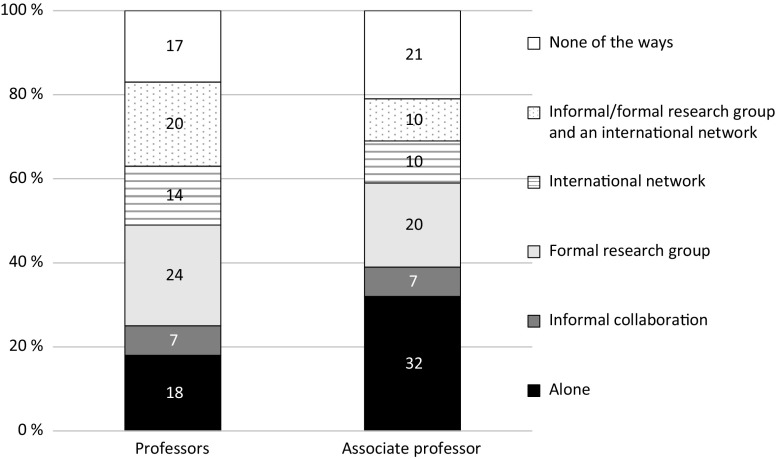
Percentages of academic staff who conduct their research in various ways “to a large degree”, by rank

Researchers above 60 years of age undertake research in international networks to a lesser extent than their younger colleagues. There are generally very small gender differences in the ways of undertaking research. The differences between associate professors and professors are however greater. Figure 1 displays that professors report to a much higher degree than associate professors that they conduct their research in formal groups and in international networks, while associate professors more often research alone.

So far we have undertaken bivariate analyses. However, logistic regression can control for different variables at the same time. Hence, we do four different logistic regressions using each of the four different ways of conducting research “to a large degree” as dependent variables, controlling for gender, age, academic rank, type of group membership, and academic field (Table 5).

**Table 5:** Logistic regressions for academic staff who conduct their research in various ways “to a large degree”

Models	1	2	3	4
Dependent variables	Alone	Formal	International network	International network in combination with a group
Gender (women = 0, male = 1)	0.549*** (0.166)	−0.0718 (0.163)	−0.305 (0.199)	0.0379 (0.186)
Age (60 years or older as ref. group)				
Less than 40 years	0.114 (0.275)	0.653* (0.286)	0.532 (0.350)	0.813* (0.349)
40–50 years	−0.161 (0.202)	0.348 (0.204)	0.408 (0.246)	0.664** (0.231)
50–60 years	−0.0401 (0.182)	0.204 (0.184)	0.0609 (0.235)	0.445* (0.208)
Member of a research group	−0.497** (0.157)	0.897*** (0.217)	−0.0108 (0.213)	0.689** (0.235)
Professor	−0.742*** (0.160)	0.142 (0.166)	0.547* (0.212)	0.944*** (0.204)
Academic field (humanities as ref. group)				
Social sciences	−0.564** (0.175)	0.673* (0.265)	−0.590* (0.259)	0.856** (0.273)
Natural sciences	−1.922*** (0.277)	1.099*** (0.275)	0.440 (0.249)	0.726* (0.296)
Technology	−1.092*** (0.234)	0.925** (0.288)	−0.483 (0.307)	0.386 (0.318)
Medicine	−2.084*** (0.289)	1.672*** (0.259)	−1.407*** (0.369)	0.657* (0.292)
Constant	−0.127 (0.231)	−3.395*** (0.332)	−2.157*** (0.313)	−4.073*** (0.377)
Observations	1385	1385	1385	1385
Pseudo *R* ^2^	0.127	0.072	0.055	0.053
Nagelkerke *R* ^2^ (without academic fields)	0.079	0.049	0.018	0.059
Nagelkerke *R* ^2^ (with academic fields)	0.189	0.109	0.074	0.075
−2 Log likelihood	1215.279	1238.094	886.071	1027.790

Standard errors in parentheses

* *p* < 0.05; ** *p* < 0.01; *** *p* < 0.001

Doing the regressions subsequently, introducing more and more variables into the model, a first striking result is how much of the variation the model explains increases when academic fields are introduced. This confirms our findings from the bivariate analyses that the importance and use of research groups differ much between the academic fields.

Table 5 shows that being a man has a significant effect on the probability to work alone. However, gender has no other significant effect. Furthermore, the younger the staff, the more they are involved in international research networks. Finally, being a professor increases the probability to work in an international network, and decreases the probability to work alone.

Although being a member of a research group does not necessarily mean that academic staff conduct their research to a great extent within the group, being a member increases the likelihood to do so. Being a member of a group also increases the probability to work in an international network in combination with a group, and decreases the probability to work alone.

Academic field is introduced into the model with the humanities as reference category. The probability to work alone is less for all other academic fields, and the probability to work in a formal research group is higher for all other fields. The probability to work in an international group in combination with a group is also higher for the social sciences, the natural sciences and medicine. These results are in line with the bivariate analyses.

Hence, rank, academic field, and whether or not academic staff are members of a research group seem to be the important factors which affect the way the academic staff conduct their research.

### Research collaboration and publication productivity

One of the purposes of the formalization of research groups is to increase the publishing activity of academic staff. Table 6 displays that group members have more publication points (3.1) than non-members (2.6). However, this difference is only significant between researchers in the social sciences, probably due to the small size of the subgroups in the various fields.

**Table 6:** Number of publication points by different types of group membership

	Humanities	Social science	Natural science	Technology	Medicine/health	Total	*N*
All	3.3	3.2	3.1	3.4	2.0	3.0	1481
Non-members	2.9	2.5	2.8	2.5	1.5	2.6	339
Members	3.6	3.4	3.2	3.6	2.1	3.1	1046
*Members who to “a large degree” conduct their research*							
Alone	4.6	3.2	0.8	3.5	0.9	3.4	174
Informal collaboration	2.4	3.9	1.6	3.0	2.3	2.7	53
Formal research group	4.0	2.8	3.4	3.4	2.1	2.9	231
International network	3.3	4.5	3.4	3.9	1.6	3.6	110
Informal/formal research group and an international network	3.8	4.4	3.9	4.6	3.1	3.9	160
None of the ways	2.6	3.2	3.1	3.5	1.8	2.9	162

The most productive group members are those who “to a large degree” undertake their research in an international network in combination with formal or informal collaboration with colleagues within the institution. This finding confirms previous studies that have found that international collaboration increases the scientific performance of research groups (Barjak and Robinson 2007; Martin-Sempere et al. 2002; Van Raan 1998) and the productivity of individual researchers (Kyvik and Larsen 1994; Abramo et al. 2011).

There are, however, clear differences across the fields. Humanists who work to “a large degree” alone have significantly more publication points than their colleagues who work to “a large degree” in an international network. Members in the social sciences who work to “a large degree” in an international network have significantly more publication points than social scientists who work “to a large degree” in a formal research group. Members in the natural sciences who conduct their research in an international network, and researchers in medicine who work in formal groups or international networks, have significantly more publication points than their colleagues who work alone. There are no such significant effects between researchers in technology, though this could be because of the small size of the subgroups (reaching from 7 to 80).

The number of publication points also varies between other variables. Men have more publication points than women. Professors are more productive than associate professors. Younger researchers tend to have more publication points than older researchers, but the difference is not significant.

As mentioned, 10% of the members do not have any publication points. Another way of investigating the difference in productivity between members and non-members is hence to see if the share of non-publishers is greater among the non-members. Only 7% of the members are non-publishers, in contrast to 20% of the non-members. Furthermore, the share of non-publishers is greatest among the researchers who work alone “to a great extent” (20%), while only 1% of the researchers that work in an international network in combination with a group “to a large extent” do not have publication points. The share of non-publishers among the researchers working in a formal research group “to a large extent” is 5%.

The share of non-publishers increases with age, from the group of researchers under 40 years of age where only 6% are non-publishers to the group of researchers over 60 where 15% are non-publishers. We find a similar difference between professors among which only 7% do not have publication points while 15% of the associate professors are non-publishers. There are no gender differences in the share of researchers that have and have not publication points.

### Research collaboration and publication quality

While the number of publication points is primarily an indicator of publication activity, the percentage of publications in prestigious journals (Level 2 publications) can be regarded as an indicator of the quality of publications (Table 7). Group members have a greater share of their publications at Level 2 (29%) than non-members (23%). However, when 43% of the researchers have no publication at Level 2 and 21% have 50%, the average share of publication at Level 2 usually become a share of publication that few researchers have. Having publications at Level 2 or not might be a better quantitative measure of the scientific output.

**Table 7:** Percentage of researchers that have publications at Level 2 by different types of group membership

	Humanities (%)	Social science (%)	Natural science (%)	Technology (%)	Medicine/health (%)	Total (%)	*N*
All	45	51	73	56	70	57	
Non-members	37	47	63	45	67	47	339
Members	51	53	75	59	71	61	1046
*Researchers who to “a large degree” conduct their research*							
Alone	54	33	21	42	40	41	174
Informal collaboration	50	45	57	45	63	53	53
Formal research group	55	55	76	65	75	68	231
International network	46	70	79	47	43	63	110
Informal/formal research group + international network	73	75	89	79	84	81	160
None of the ways	43	53	76	64	74	62	162

Table 7 displays that while five out of ten of the non-members have publications at Level 2, six out of ten of the group members have publications on this level. We find an even greater difference between researchers who work in an international network “to a large degree” in combination with a group (eight out of ten have publications at Level 2), and those who primarily research alone “to a large degree” (four out of ten).

The share of researchers having publications at Level 2 varies between background variables. While 52% of the females have publications at this level, 40% of the males have. The youngest researchers are also the group where most researchers have publications at Level 2. The greatest difference is between professors where 68% of the staff have publications at Level 2, in contrast to only 42% of the associate professors.

### Regression analyses

Table 8 displays the results of the regression analyses with researchers without and with publication points (model 1), publication points (the logarithm, model 2) and researchers without and with publications at Level 2 (model 3) as dependent variables. In these models, we control for membership in a research group, being a leader of a research group, gender, rank, age, academic field, and how group members conduct their research.^3^ We use the humanities as reference category for the same reason as in Table 4. We only include working alone and in an international network in combination with a group since these ways are the only two that have significant effects on the publication output in these models, and including only these two types of conducting research contributes to enough researchers in the reference category.

**Table 8:** OLS Regression analyses

	Publication points		Publication at Level 2
	(0 = have not, 1 = have)	The log of publication points	(0 = have not, 1 = have)
Variable names/type of regression	Logit	OLS	Logit
Gender (male = 1)	−0.132 (0.210)	0.0507 (0.0634)	0.238 (0.130)
Age (over 60)			
Under 40 years	1.684*** (0.418)	0.420*** (0.116)	1.211*** (0.240)
40–50 years	1.172*** (0.263)	0.280*** (0.0792)	0.626*** (0.163)
50–60 years	0.601** (0.216)	0.137 (0.0705)	0.331* (0.143)
Group membership (non-member)			
Ordinary member	0.645*** (0.192)	0.0811 (0.0685)	0.180 (0.134)
Group leader	1.561*** (0.371)	0.120 (0.0818)	0.638*** (0.172)
Rank (associated professor)			
Professor	1.104*** (0.200)	0.520*** (0.0656)	1.151*** (0.132)
Academic field (humanities)			
Social science	0.374 (0.237)	−0.153 (0.0827)	0.111 (0.161)
Natural science	0.712* (0.345)	−0.360*** (0.0959)	0.728*** (0.199)
Technology	−0.171 (0.279)	−0.249* (0.0996)	−0.0398 (0.193)
Medicine	0.774* (0.318)	−0.880*** (0.0912)	0.739*** (0.188)
Ways of conducting research			
Alone	−0.606** (0.200)	−0.0170 (0.0793)	−0.509*** (0.151)
International network in combination with a group	1.904** (0.727)	0.140 (0.0828)	0.776*** (0.202)
Constant	0.383 (0.277)	0.455*** (0.105)	−1.386*** (0.208)
Observations	1481	1326	1481
Pseudo *R* ^2^	0.153		0.120
Nagelkerke *R* ^2^	0.153		0.120
−2 Log likelihood	0.200		0.203
*R* ^2^		0.123	

Number of publication points as dependent variables

Standard errors in parentheses

* *p* < 0.05; ** *p* < 0.01; *** *p* < 0.001

The results in model 1 indicate that being younger than 60 years of age, being a professor instead of an associate professor, and being a researcher in the natural sciences or medicine instead of in the humanities increases the probability of having publication points. Being a man or a woman has no significant effect. Being a member of a group instead of a non-member increases the probability to have publication points, and being a leader of a group increases the probability of having publication points even more. Finally, conducting research “to a large degree” in an international network in combination with a group has the strongest effect on publication output.

Model 2 displays which factors have a significant effect on the number of publication points, where we have excluded the researchers with no publications. This is an OLS-regression where the dependent variable is the logarithm of publication points. Being a researcher in all fields but the humanities decreases the number of publications. This reflects the bias of the publication points, where researchers in the humanities have more publication points due to fewer co-authors. Also, being a researcher under 50 years of age instead of over 60 increases the number of publication points, and so does being a professor instead of an associate professor.

In model 3, being a leader of a research group, being younger than 40 years of age, being a professor, researcher in the natural sciences or medicine, and doing research in an international network increase the possibility of having publication points at Level 2. However, although collaboration in international networks has a significant effect, the age effect and the rank effect have stronger impact on the probability of having publications at Level 2.

## Discussion

While previous studies have examined separately the roles and effects of groups and networks in the scientific system and the research production, the purpose of this study has been to investigate the relative importance of research groups and research networks for individual academics.

The results of this study can be summarized as follows: At the system level, conducting research in groups in the university department and in international networks are equally common, but there are large differences between academic fields. Research groups are still more common and play a more important role in the experimental sciences than in the social sciences and above all in the humanities. The research group is clearly most important in the field of medicine and health, while undertaking research in an international network is most important in the natural sciences. However, being a member of a formal research group in the university department does not necessarily imply that the bulk of the research is done within the frame of the group; for many the participation in networks; and particularly international networks; is considered as more important.

For most academic staff, undertaking research in an international network cannot be regarded as an alternative to collaboration in the group, but as a complementary research strategy. The majority conduct their research in various ways, but with more emphasis on one way of working than another. This finding is in line with studies by Jacob and Meeks (2013), which show how researchers move to new institutions to collaborate with new colleagues, while continuing the collaboration with their previous group members.

Furthermore, we find that older academic staff are less inclined than their younger colleagues to collaborate in international research networks, and that they tend to work more alone. The tendency to less international collaboration is consistent with the tendency in two previous surveys (1992 and 2001) in Norwegian research universities. International research collaboration has increased over time, and more so among young than old academics (Kyvik and Olsen 2008). The most likely explanation is that younger academic staff are more cosmopolitan in their research orientation due to generational differences in socio-cultural influences and socialization processes at different points in time.

Membership in a research group and active participation in international research networks are likely to enhance publication productivity and the quality of publications. However, the effects as measured in this study are not very strong. Nevertheless, these results corroborate previous findings by Abramo et al. (2009) on the relationship between research collaboration and productivity, and by Andrade et al. (2009) on the relationship between collaboration in international networks and research quality. Similar to Lazega et al. (2008), we also find that group leaders appear to more productive than ordinary group members, particularly when they collaborate in international networks.

The explanatory power of our regression models is relatively low. This outcome is common in social science research, but still deserves a comment. Individual research collaborations depend on a diverse set of environmental conditions, and the research agenda can to a large extent be determined by disciplinary norms and traditions, the strategy of the research group, institutional strategies, and the priorities of funding agencies. Hence, there are many factors that affect the intensity of research collaboration in groups and networks. Likewise, individual differences in publishing productivity and the quality of the research output is dependent on a large number of factors in addition to those applied in this study.

This study is based on data from the major Norwegian research universities and has provided new information on collaborative patterns in a particular national system. As a small country in the global research community (Gornitzka and Langfeldt 2008), we might speculate whether participation in international research networks might have a more prominent role than in the larger countries due to the relatively limited number of national colleagues within the same research specialty. However, research in most countries is now increasingly undertaken in an international context of collaboration between groups and individuals (Adams 2013). Hence, we believe that the results of this study are of clear relevance to other national systems.

## Footnotes

1 We do not show the same analysis for informal collaboration, since only six percent of the respondents answered “to a large degree” on this alternative.

2 In an log-linear model where log *Y*_*i*_ = *a* + *bX*_*i*_ + *e*_*i*_, the increase of an independent variable on the dependent variable is *Y* = (e^*x*^) − 1.

3 We have tested for interaction terms between age and rank, age and gender, gender and rank. None of them had any significant effect.
